# Diagnostic Accuracy of Electrochemical Skin Conductance in the Detection of Sudomotor Fiber Loss

**DOI:** 10.3389/fneur.2020.00273

**Published:** 2020-04-17

**Authors:** Michal G. Porubcin, Peter Novak

**Affiliations:** Brigham and Women's Faulkner Hospital, Harvard Medical School, Boston, MA, United States

**Keywords:** small fiber neuropathy, skin biopsy, electrochemical skin conductance, autonomic failure, dysautonomia

## Abstract

**Background:** Small fiber neuropathy (SFN) is a common health problem. SFN is associated with loss of small fibers, either sensory, autonomic or both. Reduced autonomic sudomotor sweat gland nerve fiber density (SGNFD) and sensory epidermal nerve fiber density (ENFD) can be seen in SFN. Electrochemical skin conductance (ESC) is a non-invasive test for measurement of sudomotor function. This study evaluated the diagnostic accuracy of ESC to detect abnormal SGNFD and ENFD.

**Methods:** This was a retrospective blinded study of participants referred for evaluation of SFN. The primary outcome measure was the specificity and sensitivity of ESC to diagnose loss of small fibers using SGNFD and ENFD as reference tests. The secondary outcome measures were the correlation between ESC and neuropathy severity, pain, and autonomic clinical scales.

**Results:** Two hundred ten patients were enrolled in the study, age (mean ± sd) 45.5 ± 16.1 years, men/women = 52/158. ESC adjusted for weight (ESC/kg) was reduced in subjects with abnormally low SGNFD (normal/abnormal, ESC/kg = 1.19 ± 0.31/0.94 ± 0.37 μS/kg, *p* < 0.0001) and abnormally low ENFD (normal/abnormal ESC/kg 1.20 ± 0.37/1.04 ± 0.33 μS/kg, *p* < 0.0011). ESC/kg correlated with SGNFD (ρ = 0.39, *p* < 0.0001) and ENFD (ρ = 0.47, *p* < 0.0001). ESC/kg did not correlate with symptom scales. ESC/kg had 64% sensitivity and 77% specificity (ROC 0.73, *p* = 0.0001) to predict abnormal SGNFD and 69% sensitivity and 55% specificity (ROC 0.63, *p* = 0.0017) to predict abnormal ENFD. In comparison, SGNFD had 50.1% sensitivity and 85.1% specificity to predict abnormal ENFD (ROC 0.69, *p* = 0.0001).

**Conclusion:** ESC/kg has modest accuracy to detect SGNFD loss. ESC may be a useful test in characterization of small fiber neuropathy.

## Introduction

Small fiber neuropathy (SFN)—whether idiopathic or secondary—is common, affecting millions of people worldwide, and may be associated with considerable disability (1–5). SFN is associated with loss of small fibers, either sensory, autonomic, or both. A reliable diagnosis can be made using skin biopsy for sensory epidermal nerve fiber density (ENFD) (1, 3, 5), but skin biopsy is invasive; skin sample processing is expensive and not widely available. Electrochemical skin conductance (ESC) measurement (6) was recently introduced as a non-invasive and simple method for evaluation of sweat gland function (7, 8). Sudomotor fibers control sweat glands and the loss of sudomotor fibers correlates with ESC (9). Several studies have analyzed the diagnostic utility of ESC in detecting SFN. Most of the diagnostic studies analyzed diabetic neuropathies, and reported sensitivities/specificities were in the range of 53–88%/49–92%, depending on the particular reference test (8). Many studies applied complex clinical instruments to measure neuropathy severity and/or cardiovascular autonomic neuropathy indices as reference tests.

Since ESC measures sudomotor activity, it is desirable to assess the diagnostic accuracy of ESC using direct measures of sudomotor fiber damage such as sweat gland nerve fiber density (SGNFD) (9, 10).

In this study the diagnostic accuracy of ESC was evaluated against SGNFD as the reference test. ENFD was also used for comparison to ESC since ENFD, which is commonly employed to diagnose small fiber neuropathy, is reduced in SFN and has high diagnostic accuracy (11).

### Standard Protocol Approvals

The study was approved by the Institutional Review Board of the Brigham and Women's Hospital, Harvard Medical School, as a minimal risk study.

## Materials and Methods

### Study Design

The study design follows STARD 2015 guidelines (12). This was a retrospective, single center study.

### Participants

The study participants included consecutive subjects who were referred for evaluation of SFN to the tertiary care setting at the Autonomic Laboratory at the Brigham and Women's Faulkner (BWHF) Hospital in 2016–2017. Inclusion criteria were: (1) patients older than 17 years who had evaluation for SFN with skin biopsies for evaluation of ENFD and SGNFD; and sudomotor testing using ESC at the BWHF autonomic laboratory; and (2) availability of medical records. The records were reviewed for presence of symptoms, past medical history, and current medication. The medical records were also reviewed for the presence of large fiber neuropathy. The skin biopsies were not performed and participants were not included in this study if there was evidence of large fiber neuropathy on the neurological examination or on the nerve conduction studies. Patients were determined as having large fiber neuropathy if there was evidence of abnormal sensation for vibration and/or proprioception and/or reduced or absent deep tendon reflexes on the examination; or if the nerve conduction studies confirmed large fiber neuropathy (13). Furthermore, the medical records were reviewed for the evidence of disorders associated with SFN including diabetes, borderline diabetes, Parkinson's disease, atypical parkinsonism, history of heavy alcohol exposure, B12 deficiency, folate deficiency, thyroid disease, celiac disease, hepatitis C, HIV infection, exposure to chemotherapy, cancer, systemic autoimmune disease (SLE, Sjögren syndrome, rheumatoid arthritis, autoimmune thyroiditis, celiac disease, or other disorders associated with systemic autoimmunity) or any comorbid conditions or use of medication reported to be associated with small fiber neuropathy (5).

### Evaluation of Sensory and Autonomic Symptoms

Sensory evaluation was conducted using the Neuropathy Total Symptom Score-6 (14) and autonomic symptoms were assessed using the Survey of Autonomic Symptoms (15).

### Skin Biopsies

Skin biopsies were performed for assessment of ENFD and SGNFD according to the recommended standards (1, 3). Typically, two skin biopsy samples were obtained, one from the proximal thigh 20 cm distal to the iliac spine and the other at the calf (10 cm above the lateral malleolus) using a 3-mm circular disposable punch tool. Samples were transferred into 2% paraformaldehyde-lysine-periodic acid fixative and transported overnight to the laboratory using cold packs to maintain cooling. Skin samples were processed at a commercial laboratory (Therapath, New York, NY). Skin samples were immunoperoxidase-stained for the axonal marker PGP 9.5. Linear ENFD was determined using bright light microscopy according to the accepted guidelines (1). SGNFD was determined using the same tissue sections also stained for PGP 9.5. Images of one or two sweat glands were processed according to Gibbons et al. (16). The University of Massachusetts' lower limits of normal ENFD (fibers per millimeter of epidermal length) at the calf are 9.5–0.075^*^age for men and 11.1–0.08^*^age for women (17). The lower limit of normality for SGNFD at the calf is 36.5 fibers per cubic millimeter and was determined by Therapath.

### Sudomotor Function Testing Using ESC

ESC measurements were performed using the Sudoscan device (Impeto Medical, Paris, France) (6). Subjects placed both palms and soles on stainless steel electrodes during the 3-min scan while standing. A low direct current voltage (<4 V) was applied incrementally to the electrodes, generating a current proportional to the chloride ion flow extracted from the skin. ESC expressed as the current in microSiemens (μS) was acquired for each foot and hand. Our normative data are ≥1.03 μS/kg at hands and ≥1.14 μS/kg at feet (18).

### Statistical Analysis

For the statistical analysis, the skin biopsies from calf and the average ESC from both feet were used. A previous study (9) showed a significant correlation between ESC and skin biopsies when ESC was adjusted for weight in kilograms [ESC/kg = raw ESC data/weight (kg)]; therefore both adjusted and non-adjusted ESC were used for analysis.

ESC, ENFD and SGNFD had non-normal distribution. ESC in subjects with abnormal biopsies were compared to subjects with normal biopsies using non-parametric Wilcoxon test.

The relationships between continuous variables were obtained using Spearman's rank correlation (ρ) coefficient. Least squares (LS) models were used to evaluate the relationships between ENFD and SGNFD as a dependent variable with age, gender, and weight as model effect. Receiver operating characteristic (ROC) curves were calculated to compare the diagnostic accuracy of ESC using both SGNFD and ENFD as a reference.

Since two principal comparisons (ESC with ENFD and ESC with SGNFD) were performed, the significance was adjusted using Bonferroni corrections. For a hypothesis with a desired α = 0.05 and two comparisons, the Bonferroni-corrected α became equal to 0.025 (obtained by 0.05/2) which is required for the tests to be significant. Statistical analysis was done using JMP 13.0 (Cary, NC) statistical software.

## Results

**Figure 1:**
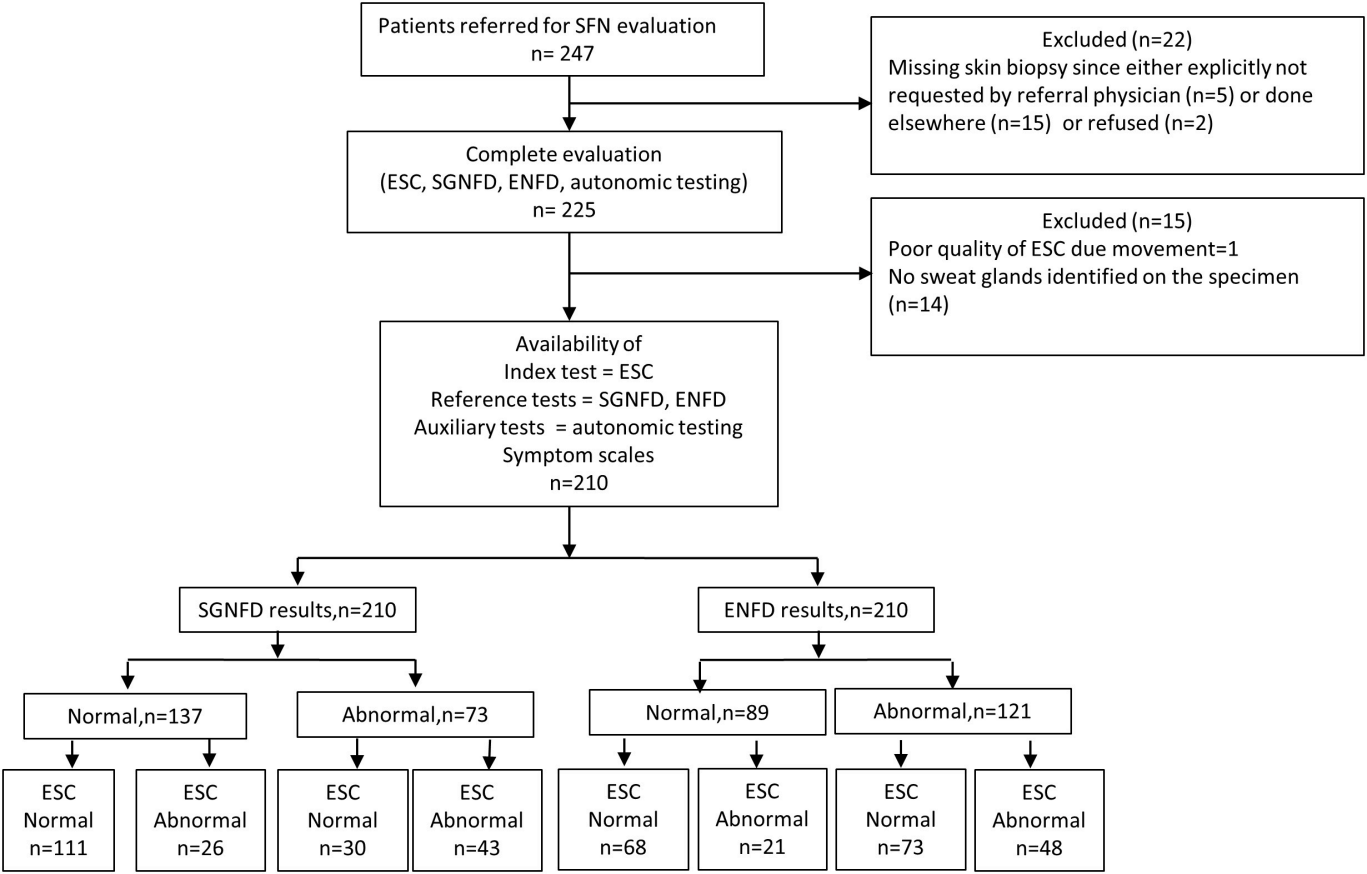
The study flow diagram.

Figure 1 shows the study flow. From a total 247 consecutive patients referred for evaluation of SFN, 210 patients were enrolled in the study [(mean ± sd): age = 45.5 ± 16.1, men/women = 52/158, BMI = 26.2 ± 6.3 kg/m^2^, Caucasian = 201, African American = 3, Hispanic = 4, Asian = 2].

Most common complaints were decreased sweating at legs (100%), orthostatic lightheadedness (90%), and aching pain (85%, Table 1). The pain during the testing was present in 82% of patients. Idiopathic SFN, was identified in 132 (63%) patients. Disorders associated with SFN were identified in 78 subjects (*n*), such as impaired glucose tolerance (2), systemic lupus erythematosus (1), thyroid disorder (6), B12 deficiency (2), Parkinson disease (1), dyslipidemia (5), metabolic syndrome (4), Sjögren syndrome (2), history of Lyme disease (30), autoimmune SFN (4), monoclonal gammopathy (2), Hodgkin lymphoma (1), non-Hodgkin lymphoma (1), exposure to chemotherapy (1), and hypermobile Ehlers-Danlos syndrome (16).

**Table 1:** Clinical complaints.

**Symptom**	***N***
Symptom duration (years, mean ± sd)	4.1 ± 3.3
Lightheadedness	190
Dry mouth or dry eyes	160
Pale or blue feet	112
Feet colder than the rest of body	158
Decreased sweating of feet at rest	210
Decreased sweating at feet after exercise or during hot weather	90
Sweating increased at hands	85
Nausea, vomiting or bloating after meal	129
Persistent diarrhea	91
Persistent constipation	112
Leaking of urine	96
Difficulty with erections (man)	33
Aching pain	179
Allodynia	82
Burning pain	125
Lancinating pain	143
Numbness	155
Prickling sensation	173
Current pain (>0)	170

None of our subjects experienced any complications from the skin biopsies and autonomic testing, or any side effect associated with use of the Sudoscan device.

SGNFD was abnormal in 73 subjects, ENFD in 121 subjects. Both SGNFD and ENFD were abnormal in 60 subjects, ENFD alone was abnormal in 61 subjects, SGNFD alone in 13 subjects, and 76 subjects had both biopsies normal. ESC was abnormal in 141 subjects (Appendix).

**Figure 2:**
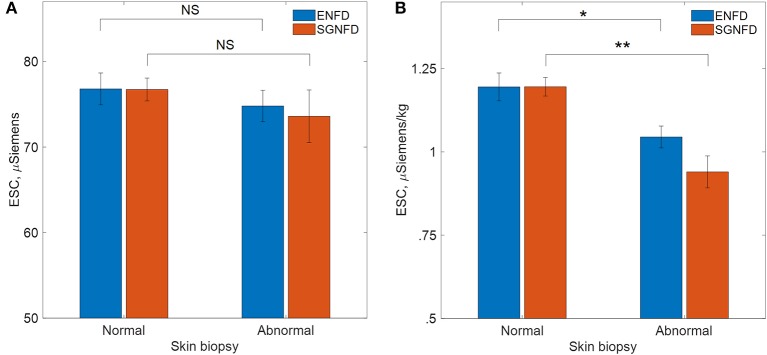
Comparison of ESC in patients with normal and abnormal skin biopsies, **(A)** μSiemens=unadjusted ESC data, **(B)** μS/kg=adjusted ESC data by weight in kg, ENFD, epidermal nerve fiber density; SGNFD, sweat gland nerve fiber density, *p < 0.001, **p < 0.0001.

There was no difference in unadjusted ESC data compared to normal vs. abnormal SGNFD (SGNFD normal/abnormal: ESC 76.7 ± 14.8/73.6 ± 18.9 μS, *p* = 0.35) or ENFD (ENFD normal/abnormal: ESC 76.8 ± 16.5/74.8 ± 16.3 μS, *p* = 0.09, Figure 2).

ESC/kg was reduced in subjects with abnormally low SGNFD (SGNFD normal/abnormal: ESC/kg = 1.19 ± 0.31/0.94 ± 0.37 μS/kg, *p* < 0.0001) and abnormally low ENFD (ENFD normal/abnormal: ESC/kg 1.19 ± 0.37/1.04 ± 0.33 μS/kg, *p* = 0.001, Figure 2).

ESC correlated with ENFD (ρ = 0.30, *p* < 0.0001) but not with SGNFD (ρ = 0.08, *p* < 0.26). ESC did not correlate with shoe size (ρ = 0.05, *p* < 0.53) and with weight (ρ = −0.03, *p* < 0.67). ESC/kg correlated with SGNFD (ρ = 0.39, *p* < 0.0001) and ENFD (ρ = 0.47, *p* < 0.0001). ESC and ESC/kg did not correlate with the Survey of Autonomic Symptoms or the Neuropathy Total Symptom Score-6.

**Figure 3:**
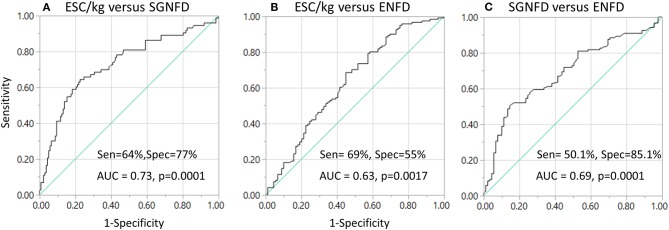
Sensitivity and specificity of ESC in detection of small fiber loss **(A,B)** using ROC analysis and skin biopsies as reference tests. Sensitivity and specificity of SGNFD to detect abnormal ENFD **(C)**. AUC, area under the curve; Sen, sensitivity; Spec, specificity.

Using ROC analysis, ESC does not predict abnormal SGNFD (ROC area 0.54, *p* = 0.2) or ENFD (ROC area 0.57, *p* = 0.4, Figure 3). ESC/kg had 64.4% sensitivity and 77.4% specificity (ROC area 0.73, *p* < 0.0001) to predict abnormal SGNFD and 68.6% sensitivity and 55.1% specificity (ROC area 0.63, *p* = 0.0017) to predict abnormal ENFD (Figure 3). SGNFD predicted abnormal ENFD with a ROC area of 0.69 (*p* = 0.0001, 50.1% sensitivity and 85.1% specificity).

## Discussion

This study evaluated diagnostic accuracy of ESC to detect loss of small fibers. SGNFD and EFND were used as reference tests. ESC adjusted for weight (ESC/kg) correlates with SGNFD and ENFD. The highest diagnostic accuracy was achieved when ESC/kg was compared to SGNFD (ROC = 0.73, sensitivity 64%, specificity 77%). When comparing ESC/kg to ENFD, the ROC and specificity were lower although sensitivity was slightly higher (ROC = 0.63, sensitivity 69%, and specificity 55%). For comparison, ENFD predicted SGNFD loss with relatively high specificity (82.5%) but the sensitivity was low (50%).

In terms of ROC, there was more proximity between ESC and SGNFD than between ESC and ENFD, supporting the notion that ESC and SGNFD assess similar types of fibers, i.e., sudomotor autonomic. The modest correlation between SGNFD and ESC is not surprising since SGNFD and ESC were collected at different sites (calf vs. sole) and are different measures (numeric fiber count vs. functional conductance).

This study replicated several results from our previous study (9) as well as another study (19) using a separate patient population and different laboratory. Again, the highest correlation between ESC and skin biopsies was achieved by adjusting ESC for weight. The previous study (9) showed moderate correlations between ESC and SGNFD (ρ = 0.64) as well as ENFD (ρ = 0.73**)** that were similar to this study.

In this study ESC was compared to both ENFD and SGNFD. ENFD is a reliable marker of small fiber neuropathy with high diagnostic accuracy and reproducibility which have been confirmed in many studies, with sensitivities and specificities in the range of 78–92% and 65–90%, respectively (4). Although the accuracy of SGNFD in the diagnosis of small fiber neuropathy is unclear, SGNFD is reduced in diabetes and correlates with clinical measures of neuropathy severity (16). Furthermore, it can be argued that SGNFD obtained from one or two sweat glands may not be representative of a global sudomotor nerve density. However, serial sections of skin biopsies showed low intersection variability (variance 13–38% among sweat gland sections from the same biopsy sample) indicating that the sufficient sampling can be provided from low number of sweat glands (16).

The sensitivity of ESC to detect SGNFD or ENFD is not high enough to replace SGNFD or ENFD. Particularly ESC has very low specificity (55%) to detect ENFD loss. However, ESC reflects the cumulative function of thousands of sweat glands while skin biopsies count 1–2 sweat glands fibers at the biopsy site (3 mm wide). Furthermore, ESC measures nerve function on the soles while skin biopsies are taken from the calf. These differences in location and sampling areas may play a role in the diagnostic accuracy of ESC when compared to skin biopsies. In addition, ESC acquired at hands was not evaluated in this study.

The reason why adjustment for weight increases the diagnostic yield of ESC remains to be clarified. As discussed previously (9), potential causes include statistical reasons (adjustment changes the distribution of ESC), or differences in sampling area among subjects since the electrodes are larger than the plantar surface while foot size varies among subjects. Larger feet may produce larger sweat output, and plantar surface area is known to correlate with weight (20). By adjusting ESC for weight (dividing ESC by kilogram), the ESC/kg is in fact adjusted also for the size of the plantar surface since weight and plantar surface are correlated (*r* = 0.792, *p* < 0.001) (20).

This study showed no correlation between ESC and patients' weights. The fact that ESC adjusted for weight correlated with skin biopsies, but ESC does not correlate with weight, raises the possibility that skin biopsies correlate with weight. Indeed, a recent normative study (*n* = 550 healthy subjects, with weight available in 247 subjects) did find a correlation between ENFD and weight (R^2^ = 0.12, *p* < 0.01) in men but not in women (2). We evaluated correlations between skin biopsies and weight using pooled data from our previous studies (9, 17) since there were few males in this study. Least squares (LS) models showed significant effects of weight (R^2^ = 0.06/0.05 men/women, *p* < 0.0001) and gender (R^2^ = 0.14, *p* < 0.0001) but not age (*p* < 0.29/0.42 men/women) for SGNFD (*n* = 807, normal SGNFD in 354 subjects). LS models also showed significant effects of weight (R^2^ = 0.07/0.07 men/women, *p* < 0.0001), gender (R^2^ = 0.08, *p* < 0.0001), and age (R^2^ = 0.21, *p* < 0.0001) for ENFD (*n* = 813, normal ENFD in 354 subjects). Similar significance was achieved when calculating LS separately for normal and abnormal subjects. The above calculations indicate that weight has a significant effect on both ENFD and SGNFD. The dependence of ENFD and SGNFD on weight may explain why adjustment for weight improves correlations with ESC. Even though the effect is low [6% and 7% of variability for SGNFD and ENFD, respectively, was due to weight in this study, but 12% of variability for ENFD due to weight for men was found in the international normative study (2)], the effect may be strong enough to affect the diagnostic accuracy of ESC.

## Study Limitations

The study was retrospective, and referral bias as well as other biases may play a role in results. It should be emphasized that this study did not assess the diagnostic accuracy of ESC to detect SFN but it analyzed the diagnostic accuracy of ESC to detect SGNFD and ENFD loss. Nevertheless, the loss of ENFD is a major criterion for the diagnosis of SFN (11). Another limitation is the fact that the site of skin biopsy and of the ESC electrodes differed; hence different sudomotor fibers were assessed.

## Conclusions

This study expands our understanding of the role of ESC in the evaluation of SFN. The modest correlations among all three tests suggest that, in a cohort of symptomatic patients referred for SFN assessment, a small fiber anatomical test (ENFD) and an autonomic test (ESC or SGNFD) independently contribute to the characterization of the underlying neuropathy pathology.

## Data Availability Statement

All datasets generated for this study are included in the article/Supplementary Material.

## Ethics Statement

The studies involving human participants were reviewed and approved by Institutional Review Board of the Brigham and Women's Hospital, Harvard Medical School. Written informed consent for participation was not required for this study in accordance with the national legislation and the institutional requirements.

## Author Contributions

MP conducted analysis of the data, interpretation of results, and wrote the manuscript. PN supervised the project, designed the study, conducted the data analysis and results interpretation, and wrote the manuscript.

## Conflict of Interest

The study was supported in part by Impeto Medical, Paris, France. PN is advisor/independent contractor for Dysimmune Diseases Foundation, Panama City Beach, FL, he received speaker's honoraria from KabaFusion, Cerritos, CA; Lundbeck, Copenhagen, Denmark, and he is on scientific board of Endonovo Therapeutics, Woodland Hills, CA. The remaining author declares that the research was conducted in the absence of any commercial or financial relationships that could be construed as a potential conflict of interest.

## Supplementary Material

Appendix Individual data. Each line represent data from one subject.
